# Congenital heart malformations induced by hemodynamic altering surgical interventions

**DOI:** 10.3389/fphys.2014.00287

**Published:** 2014-08-01

**Authors:** Madeline Midgett, Sandra Rugonyi

**Affiliations:** Department of Biomedical Engineering and Knight Cardiovascular Institute, Center for Developmental Health, Oregon Health and Science UniversityPortland, OR, USA

**Keywords:** congenital heart defects, hemodynamics, vitelline vein ligation, left atrial ligation, outflow tract banding, mechanotransduction

## Abstract

Embryonic heart formation results from a dynamic interplay between genetic and environmental factors. Blood flow during early embryonic stages plays a critical role in heart development, as interactions between flow and cardiac tissues generate biomechanical forces that modulate cardiac growth and remodeling. Normal hemodynamic conditions are essential for proper cardiac development, while altered blood flow induced by surgical manipulations in animal models result in heart defects similar to those seen in humans with congenital heart disease. This review compares the altered hemodynamics, changes in tissue properties, and cardiac defects reported after common surgical interventions that alter hemodynamics in the early chick embryo, and shows that interventions produce a wide spectrum of cardiac defects. Vitelline vein ligation and left atrial ligation decrease blood pressure and flow; and outflow tract banding increases blood pressure and flow velocities. These three surgical interventions result in many of the same cardiac defects, which indicate that the altered hemodynamics interfere with common looping, septation and valve formation processes that occur after intervention and that shape the four-chambered heart. While many similar defects develop after the interventions, the varying degrees of hemodynamic load alteration among the three interventions also result in varying incidence and severity of cardiac defects, indicating that the hemodynamic modulation of cardiac developmental processes is strongly dependent on hemodynamic load.

## Introduction

Hemodynamics play an important role in regulating early cardiovascular development (Culver and Dickinson, 2010). Numerous studies have shown that surgically altered blood flow conditions in embryos result in a spectrum of cardiac defects that resemble those found in human babies with congenital heart disease (Rychter, 1962; Oscar, 1965; Harh et al., 1973; Clark and Rosenquist, 1978; Clark et al., 1984; Icardo, 1996; Hogers et al., 1997, 1999; Broekhuizen et al., 1999; Sedmera et al., 1999, 2002; Tobita and Keller, 2000; Tobita et al., 2005; Dealmeida et al., 2007; Hu et al., 2009). During normal development, cardiac performance progressively improves to meet the rising metabolic demands of the growing embryo under increased hemodynamic loading conditions (Hu and Clark, 1989). Hemodynamic loads on cardiac tissues, which are blood pressures and wall shear stresses exerted by blood flow, modulate cardiac development and are required for proper heart formation (Sedmera et al., 1999; Fisher et al., 2001; Hove et al., 2003; Van Der Heiden et al., 2006). When hemodynamic parameters are altered by surgical manipulations, normal cardiac morphogenesis is disrupted and this disruption results in cardiac malformations/defects. Cardiac anomalies observed after surgical manipulations that alter blood flow conditions in embryonic animal models are thought to result from incorrect looping, septation and valve formation, as well as improper growth and remodeling during heart development. Despite the undisputable importance of blood flow conditions on cardiac development, the mechanisms by which hemodynamic forces lead to malformations seen in congenital heart disease remain unclear.

The embryonic cardiovascular system is dramatically affected by changes in hemodynamic load. The embryonic heart, the main driver of hemodynamic loads, acutely adapts passive ventricular properties and contractile function in response to altered loads (Keller, 2001; Tobita et al., 2002; Shi et al., 2013), and proceeds to undergo abnormal growth and morphogenesis that later result in cardiac defects (Harh et al., 1973; Clark et al., 1989; Hogers et al., 1997; Sedmera et al., 1999; Tobita and Keller, 2000). Studies have shown that mechanical stresses and strains in the myocardium regulate ventricular growth and remodeling, and trigger endothelial cell organization and signaling (Fisher et al., 2001; Hove et al., 2003; Van Der Heiden et al., 2006), as well as changes in geometry and passive properties to offset the effects of altered wall stresses and/or strain (Omens, 1998). Changes in wall shear stresses, which result from changes in blood flow velocities, have also been shown to alter endocardial cell expression of shear-responsive genes (Groenendijk et al., 2007), which are important for proper endothelial response and further cardiovascular development. Altered hemodynamic conditions are tightly linked to cardiac defects: cardiac malformations not only occur due to altered blood flow, but also cardiac anomalies unrelated to altered blood flow (e.g., due to genetic mutations, teratogens, etc) affect hemodynamics and are responsible for secondary cardiovascular malformations beyond those of the original cardiac defect.

Several embryonic animal models have been used to study cardiac development and better understand the origins of congenital heart disease. Vertebrate species, in particular, are favorite models since developmental processes are highly conserved among them. Typically studied models include the mouse, the zebra fish, and the chicken (or in general avian) embryos. Due to the availability of knockouts, mouse models are typically used to assess the effects of genes on cardiac development and cardiovascular disease (Phoon et al., 2004; Bruneau, 2008). Further, because mice are mammals, mouse models are also used to assess the effects of mother's nutrition or cardiovascular conditions, as well as placental development, on embryonic growth and cardiac formation (James et al., 1998; Zhou et al., 2002; Yu et al., 2008). Mouse embryos, however, are not ideal models to study the effects of hemodynamics on cardiac development because they are very difficult to image and manipulate inside their mother's womb, and cannot develop beyond early stages outside the womb (Piliszek et al., 2011). Zebra fish and avian embryonic models have therefore been more extensively used to monitor hemodynamic conditions during development (Hove et al., 2003; Martinsen, 2005; Jenkins et al., 2010; Miura and Yelon, 2011). Chick embryos, in particular, are often used as a biological model of cardiac development because of ease of accessibility in the egg, including easy access for surgical manipulations to alter blood flow, and developmental similarities with human embryos (including the formation of a four-chamber heart). The availability of advanced *in vivo* imaging techniques (such as optical coherence tomography and ultrasound biomicroscopy) has allowed access to measuring and monitoring embryonic hemodynamic conditions over developmental stages, and to assess acute changes in hemodynamics after interventions that alter blood flow conditions (McQuinn et al., 2007; Rugonyi et al., 2008; Hu et al., 2009; Liu et al., 2012; Peterson et al., 2012). The chicken embryo has therefore been extensively used to study the effects of hemodynamic alterations on cardiac development.

**Figure 1:**
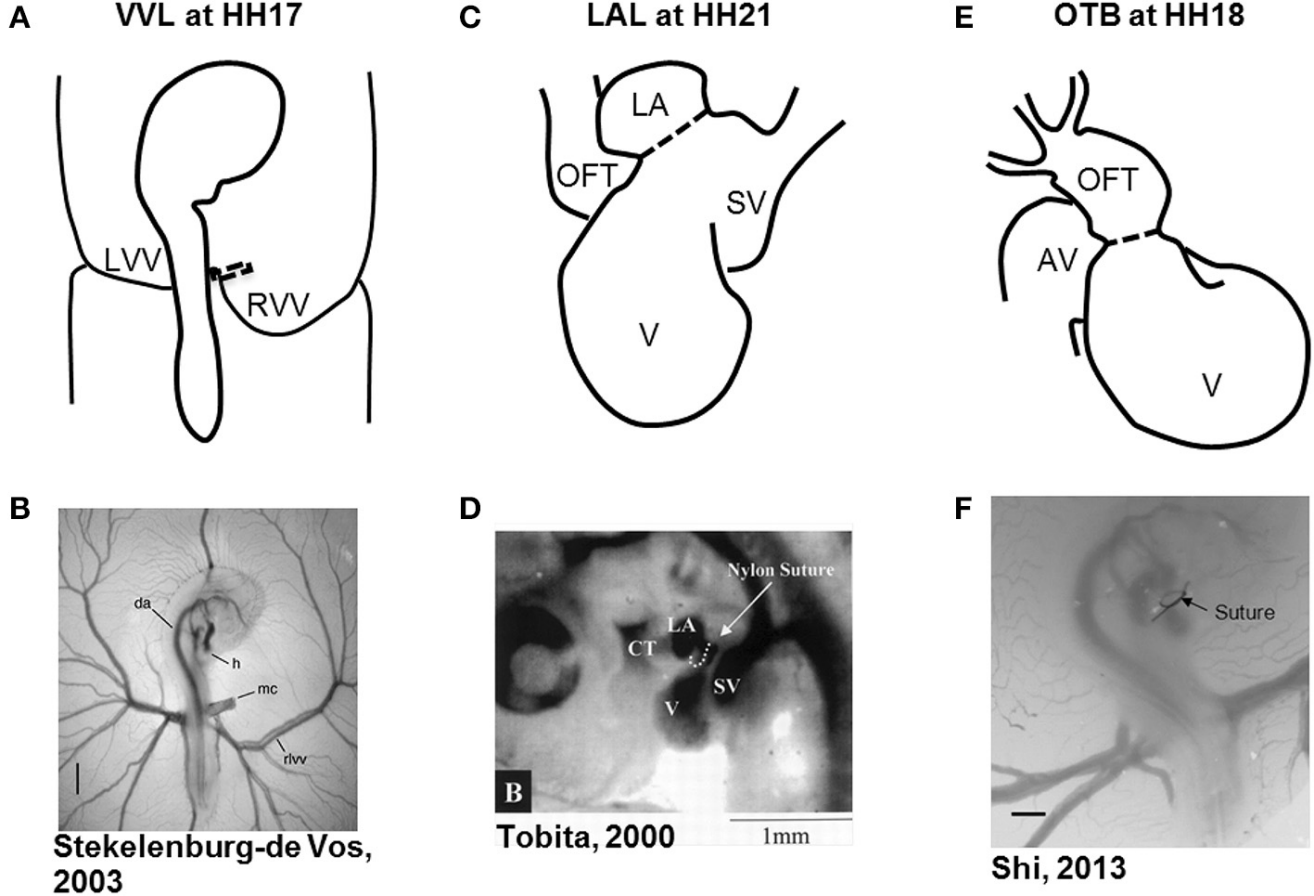
**Schematic and experimental photos of the three surgical interventions. (A,B)** right VVL at HH17. The dotted line represents the point of manipulation where either a clip or suture obstructs the flow of blood back to the heart. **(C,D)** LAL at HH21. The dotted line shows where the suture is tied around the left atrium to reduce its size and restrict flow. **(E,F)** OTB at HH18. The dotted line represents where the suture is tied around the OFT to reduce its cross-sectional area. AV, atrioventricular canal; CT, conotruncus, or heart outflow tract; DA, dorsal aorta; H, heart; LA, left atrium; LVV, left vitelline vein; MC, microclip; OFT, outflow tract; RLVV, right lateral vitelline vein or RVV; RVV, right vitelline vein, SV, sinous venosus; V, primitive ventricle. Image reproduced with permission from Tobita and Keller (2000); Stekelenburg-De Vos et al. (2003); Shi et al. (2013).

Hemodynamic interventions in the chick embryo are designed to alter preload, afterload, and blood flow volume and velocities. The three main surgical cardiac interventions investigated in the chick embryo are vitelline vein ligation (VVL), left atrial ligation (LAL), and outflow tract banding (OTB), and are summarized in Figure 1. After intervention, the induced hemodynamic alterations produce changed heart wall mechanical properties and a spectrum of cardiac defects following the completion of septation and valve formation. The three hemodynamic manipulations result in similar defects, with varying degrees of incidence and severity. VVL redirects the path of blood flow and briefly decreases hemodynamic load on the heart (Broekhuizen et al., 1999; Stekelenburg-De Vos et al., 2003, 2005, 2007; Ursem et al., 2004). LAL restricts the blood flow entering the primitive ventricle effectively reducing hemodynamic load on the heart. As the heart develops into chambers, decreased load and delayed development on the left side of the heart is compensated by the right side of the heart, leading to left heart hypoplasia, and other associated defects (Hu and Clark, 1989; Tobita and Keller, 2000; Tobita et al., 2002; Lucitti et al., 2005; Hu et al., 2009). OTB constricts the diameter of the heart outflow tract, and increases the hemodynamic load on the heart generating high-shear and high-pressure flow conditions (Clark et al., 1989; Tobita et al., 2002; McQuinn et al., 2007; Shi et al., 2013). OTB produces some of the largest incidences of defects, as well as the most severe malformations among the surgical procedures including double outlet right ventricle and persistent truncus arteriosus.

In this review, we summarize the normal heart formation process, and clinical significance of congenital heart defects that have been reported after hemodynamic interventions. We will mainly focus on research performed on avian embryos, as they are the favorite model in studying the effects of altered blood flow conditions, and thus extensive studies and available data can be easily compared. We will thus summarize the reported hemodynamic response after surgical interventions, altered cardiac mechanical properties observed, and the final defects of the four-chambered heart after VVL, LAL, and OTB in the chick embryo.

## Normal heart formation

The cardiovasculature is one of the earliest systems to form in the embryo, starting with just very thin walls that then grow and remodel over the course of development. Most of the heart morphogenesis processes occur under blood flow conditions, implying an intrinsic interaction between cardiac development and hemodynamics (Oppenheimer-Dekker et al., 1981; Icardo, 1996; Manner, 2000; Hove et al., 2003; Martinsen, 2005). In this review, we describe the initial heart tube morphogenesis into the four-chambered mature heart focusing on the developmental processes that are influenced by hemodynamics.

### Heart tube and looping

**Figure 2:**
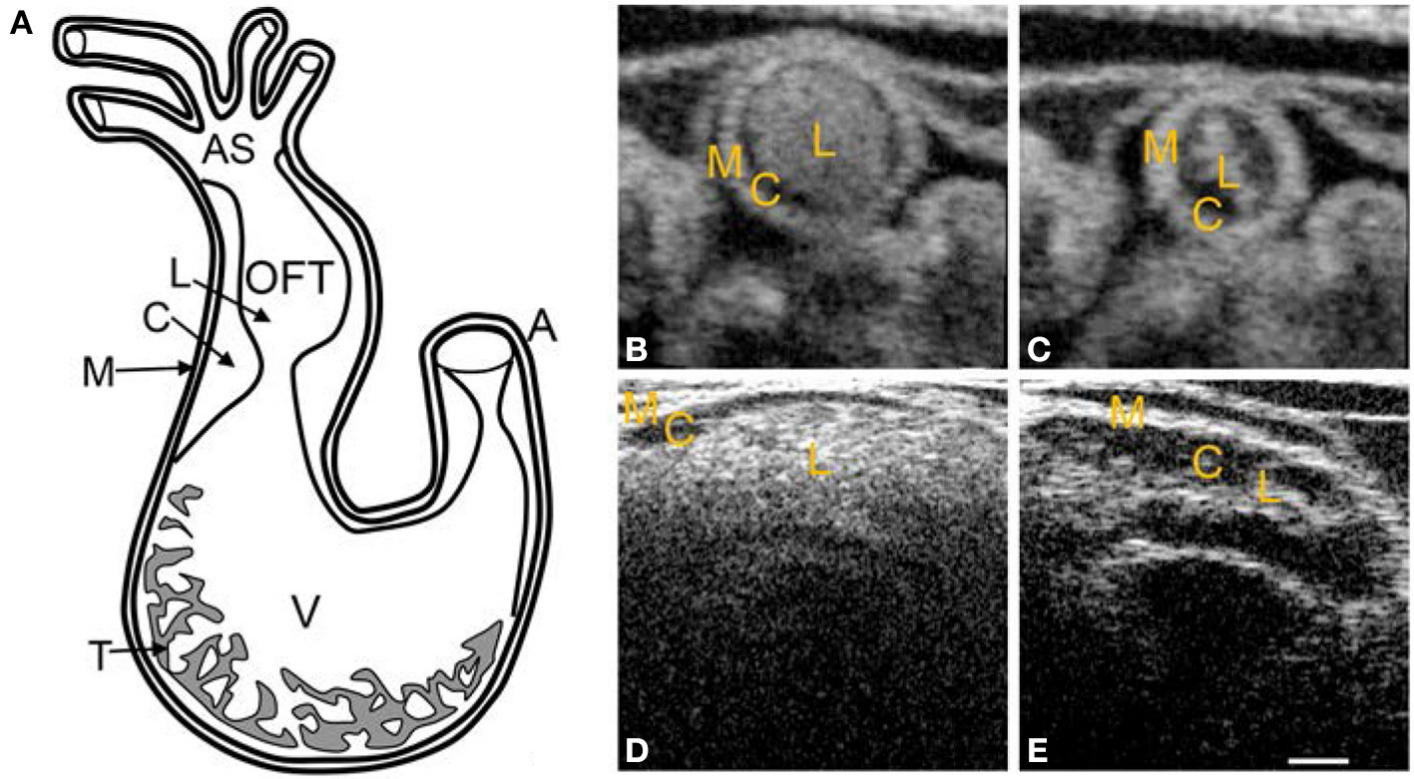
**Chick heart structure at HH18. (A)** Heart schematic showing the outflow tract cushions at the inlet of the outflow tract. **(B–E)** Representative optical coherence tomography images of the outflow tract. Cardiac jelly appears darker in between the lighter myocardium and lumen. **(B)** Cross-sectional 2D image at maximal expansion. **(C)** Cross-sectional 2D image at maximal contraction. **(D)** Longitudinal 2D image at maximal expansion. **(E)** Longitudinal 2D image at maximal contraction. A, atrium; AS, aortic sac; C, cardiac jelly; M, myocardium; L, lumen; T, trabeculae; V, ventricle. Scale bar = 200 microns.

Initial heart tube formation starts after fusion of the paired primordia at Hamburger and Hamilton (HH) stage 9 (Hamburger and Hamilton, 1992), after which the heart tube is formed down the embryonic midline. The primary heart field is the first wave of mesodermal cells that form the initial heart tube. The second heart field additionally contributes to the arterial and venous poles of the heart tube, consisting of progenitor cells that give rise to myocardium, smooth muscle cells, and endothelial cells (Kelly et al., 2001; Mjaatvedt et al., 2001; Waldo et al., 2001; Dyer and Kirby, 2009; Kelly, 2012). The early heart tube is composed of three tissue layers: the outer myocardium layer, the inner monolayer of endocardium, and a thick middle layer of largely extracellular matrix, also known as the cardiac jelly. Looping begins at HH9-10, where the initially straight cardiac tube bulges, rotates to the right, and wrap into a loop (De La Cruz et al., 1989; Icardo, 1996; Manner, 2000). After the primitive ventricular region forms a bend in the c-shaped loop at HH11–12, three morphologically distinct cardiac regions emerge: the primitive atrium, the primitive ventricle, and the primitive outflow tract (Manner, 2000). The heart tube coordinates contractions without the mature pacemaking and conduction system as early as HH10 (Martinsen, 2005). The simple c-shaped loop continues to change direction and position until it transforms into an s-shaped loop that is complete by HH24, in which the outflow tract moves over the atrioventricular (AV) canal (Manner, 2000; Martinsen, 2005). Many groups have investigated the mechanisms driving the looping process, and have presented evidence suggesting that looping is mediated by a combination of asymmetric expression of genetic and molecular markers (Mercola and Levin, 2001; Baker et al., 2008), intrinsic forces from differential growth of the heart tube (Stalsberg, 1970; Taber, 1998), and extrinsic forces of neighboring tissues (Voronov and Taber, 2002; Voronov et al., 2004). Throughout the looping process, the outflow tract and AV canal develop endocardial cushions, which are regional wall thickenings of the cardiac jelly layer, as depicted in Figure 2. Cardiac cushions serve as primitive valves that block blood flow upon contraction of the myocardium by closing the lumen. Endocardial cushions play an important role on later valve and septa formation.

Trabeculation is initiated in the primitive ventricle during the late stages of looping. Initial trabeculation starts when the cardiac jelly is displaced from the outer curvature of the ventricle and endocardial pouches grow toward the myocardium layer, creating a fine trabecular spongy network with small intertrabecular spaces (Ben-Shachar et al., 1985; Sedmera et al., 1997; Taber, 1998; Manner et al., 2009), see Figure 2. Because trabecular density increases with load, trabeculae have been linked to aiding in cardiac contraction and ventricular output flow direction. Further, because trabeculation increases ventricular luminal surface area, it has been associated to increase in the passive diffusion of oxygen to the myocardial tissue (Challice and Viragh, 1974; Taber, 1991; Yang et al., 1994). Trabeculae are important components of early heart development and later chamber formation.

The transitional zones between the primitive regions that emerge during cardiac looping are of interest concerning congenital heart malformations. These areas are brought closer together during the looping process to eventually form portions of the septa, valves, and fibrous heart matrix (Gittenberger-De Groot et al., 2005). Most surgical interventions discussed in this paper are performed at the later stages of looping (HH17–21) and therefore affect subsequent septation and valve formation and the late stages of looping.

### Pharyngeal arch artery (PAA) formation

The primitive arterial system develops through vasculogenesis, angiogenesis, and connection with the cardiogenic plates until it eventually transforms into the mature arterial system. Initially, the heart tube connects to the bilateral dorsal aortae through the first pair of PAA. During chick embryo development, a total of six pairs of PAA emerge, remodel, and transform into the mature arteries. Initial formation of the six-paired PAA in the chick embryo is similar to that of human embryos, but the involution pattern is different. In both human and chick embryos, the mature brachiocephalic arteries are derived from the third pair of arches, the aortic arch is derived from the right fourth arch, while the pulmonary arteries and the ductus arteriosus are derived from the sixth arch pair (Gittenberger-De Groot et al., 2005; Wang et al., 2009). The fifth arch pair have been have been termed to describe arteries that are only temporarily present in early development of the chick embryo, and are not considered a major PAA (Hiruma and Hirakow, 1995). Proper arch formation depends on programmed processes but is also regulated by blood flow conditions (Keller et al., 2007; Pekkan et al., 2008; Kowalski et al., 2013).

### Septation and chamber formation

Subsequent to looping, internal septa divisions and valve leaflets develop in the primitive atrial and ventricular chambers, the outflow tract, and the AV canal between stages HH21-36 (Martinsen, 2005). The individual septa join in the center of the heart to complete the transformation into a four-chambered heart. Correct looping is essential for normal cardiac septation. Septation occurs via combinations of merging and fusion of endocardial cushions, compaction of trabeculae, and ballooning of the ventricles (Icardo, 1996; Lamers and Moorman, 2002; Gittenberger-De Groot et al., 2005; Van Den Berg and Moorman, 2009).

Right and left AV orifices are created once the AV cushions meet and fuse, and eventually transform into the mitral and tricuspid AV valves. The atrial septum starts forming and grows from the atrial roof downward to merge with the AV cushions. Final closure of the atrial septum occurs shortly after birth/hatching (Hendrix and Morse, 1977). The interventricular septum develops as a common wall between the primitive right and left ventricles by merging and compaction of ventricular outer curvature trabeculae. The aorto-pulmonary septum (mesenchymal tissue) originates between the fourth and sixth PAA, divides the aortic sac, and extends and fuses to the right and left ventricles so that each communicate exclusively with the designated artery. The upstream portion of the outflow tract including the cushions are transformed into the ventricular outflows and the aortic and pulmonary semilunar valves, while the downstream portion of the outflow tract is remodeled to contribute to the proximal portion of the aorta and pulmonary trunks (Rychter, 1981; Icardo, 1996; Lamers and Moorman, 2002).

## Common heart defects following hemodynamic interventions

Nearly 1% babies born have some form of a congenital heart defect (Hoffman and Kaplan, 2002). Heart defects found in humans are similar to defects that develop after surgically altering embryonic hemodynamic conditions in animal models. In this section of the review we will summarize the definitions, prevalence, and current treatment options in human patients, of the heart defects that have been observed after hemodynamic manipulations in chicken embryos.

### Ventricular septal defects (VSD)

Ventricular septal defect (VSD) is the most common cardiac defect, occurring in 40–50% of all babies with congenital heart disease (Hoffman, 1995a; Minette and Sahn, 2006). VSD is a cardiac abnormality in which there is a hole in the ventricular septum that separates the left and right ventricles. VSD is classified by the muscular/fibrous location of the defect within the septum, and its size. Communication between the ventricles caused by VSD forces oxygen-rich blood from the left side of the heart through the defect to mix with the oxygen-poor blood from the right ventricle, with corresponding right ventricle volume overload. The presence of a VSD can result in pulmonary hypertension (from having more blood to be pumped to the pulmonary vessels) and can force the left ventricle to work harder to pump the needed blood to the body. VSD can also cause difficult breathing and heart murmurs (Minette and Sahn, 2006; Penny and Vick, 2011).

Many factors influence the hemodynamic significance of VSDs, including VSD size, ventricular pressures, and pulmonary resistance. Doppler echocardiography can diagnose VSD early in development, but sometimes VSDs reduce in size or disappear shortly after birth (Hoffman, 1995a,b). If the VSD is small enough, it can close on its own without surgery. In addition, small VSDs can be asymptomatic and not be discovered until later in life, without significantly affecting an individual's health. If the VSD is large, surgical closure is necessary. To close a VSD a patch of fabric or pericardium is sewn over the hole, and eventually the patch gets covered by normal heart tissue. Catheter closure has also emerged as an attractive VSD treatment method (Arora et al., 2004; Fu et al., 2006). Time of repair depends on the size and location of the VSD.

### Pharyngeal arch artery malformation

Many different malformations of the six-paired PAA can occur. Cardiac developmental progression establishes flow patterns through the heart that are directed into the developing PAA. The altered hemodynamics following surgical interventions also disturb aortic blood flow, which affect normal muscularization patterns and morphogenesis of the aorta. Anomalies of the aortic arch are often indicators of other congenital cardiovascular diseases (Türkvatan et al., 2009; Kellenberger, 2010).

Common cardiac defects associated with arch malformations include: double aortic arch, interrupted aortic arch, patent ductus arteriosus, and persistent truncus arteriosus. Double aortic arch is rare and occurs when the ascending aorta divides into two arch arteries, which funnel into the descending aorta. Because one of the aortic arches is close to the trachea and/or esophagus, symptoms are associated with trachea/esophagus compression (Gross, 1955). Surgery is performed in symptomatic patients.

Interrupted aortic arch is also a rare condition that occurs when there is regression of both of the right and left fourth aortic arches, and as a result the ascending and descending aorta are not connected (Kellenberger, 2010). Because an interrupted aortic arch affects blood flow to the lower body, it must be treated with surgery soon after birth.

Patent ductus arteriosus (PDA) occurs when the ductus arteriosus that typically closes after birth does not close, resulting in abnormal blood flow between the aorta and pulmonary arteries (Schneider and Moore, 2006). Incidence rates range from 1 in 500 births (Lloyd and Beekman, 1994) to 1 in 2000 births (accounting for about 5–10% of all congenital heart defects) (Mitchell et al., 1971), varying as some reports choose to include clinically silent cases that are discovered incidentally. The ductus arteriosus, which connects the aorta with the pulmonary artery, contains cushions made up of elastic fibers and smooth muscle cells that normally close at the junction of the aorta after (Oppenheimer-Dekker et al., 1981; Slomp et al., 1992). When it fails to close, the opening permits oxygen-rich blood from the aorta to mix with oxygen-poor blood from the pulmonary artery. This can strain the heart and increase blood pressure in the pulmonary arteries causing pulmonary hypertension. Sometimes the PDA closes on its own, but if it remains open, a transcatheter closing device is used. Surgical ligation and surgical division is performed when all other options have failed (Schneider and Moore, 2006).

Persistent truncus arteriosus (Arora et al.) occurs when the truncus arteriosus fails to properly divide into the pulmonary trunk and aorta. Thus instead of having outlets from the right and left ventricles, one great vessel arises above a large VSD connecting the ventricles (Marshall, 1943). PTA is rare but severe, and accounts for approximately 1% of congenital heart disease cases (Ferdman and Singh, 2003). Blood flow in the PTA goes to both the body and the lungs. Similar to VSD defects, extra blood is pumped back to the lungs, causing the left side of the heart to work harder with the risk of pulmonary hypertension. Surgery is required to close the VSD and separate the body and lung blood flow. Pulmonary arteries are removed from the great vessel and then connected to a tube with a valve inserted into the right ventricle. The tube and valve need replacement 2–3 times in childhood and into adulthood as needed.

### Atrio-ventricular and semilunar valve malformations

Valve morphology abnormalities can result in valve insufficiency and stenosis (Rapaport, 1975). Valve insufficiency occurs when the abnormal shape of the valve leaflets prevents the valve from properly closing and allows blood flow regurgitation back across the valve, causing cardiac overload. Valvular stenosis happens when the valvular tissue thickens and becomes more fibrous and stiffer, which narrows the valve opening and reduces blood flow. The fibrous valve interferes with blood flow and produces an increase in blood pressures throughout the cardiovascular system. Bicuspid aortic valve is a common semilunar valve defect, affecting 1–2% of the population, where there are only two complete commissures (Hoffman and Kaplan, 2002). AV valve anomalies are associated with aneurysms of the ascending aorta, VSD, and aortic coarctation (Kappetein et al., 1991; Arrington et al., 2008).

Mitral valve prolapse is a common atrio-ventricular valve defect, where a mitral valve leaflet is displaced into the left atrium during systole (Devereux et al., 1987; Perloff and Child, 1987). This arises when blood flow interacts with an abnormally thickened leaflet, and has been estimated to affect 2–3% of the population (Freed et al., 1999). Mitral valve prolapse is associated with mid-systolic clicks, late systolic murmurs, and other serious complications including severe mitral regurgitation and bacterial endocarditis (Devereux et al., 1989). Symptoms associated with valve insufficiency and stenosis greatly depend on the degree of flow obstruction. Patients can remain asymptomatic for years, but once they become symptomatic rapid detrimental cardiac progression begins, and surgery is recommended. Surgical interventions include repair and reconstruction or valve replacement.

### Double outlet right ventricle (DORV)

Double outlet right ventricle (DORV) accounts for approximately 1.5–2.0% of all congenital heart defects (0.03–0.1/1000 live births) (Corno, 2004). DORV encompasses a spectrum of malformations where the pulmonary artery and the aorta both arise from the right ventricle. This anomalous cardiac morphology decreases the amount of oxygenated blood that reaches the body. DORV is also always combined with VSD (Kurosawa and Becker, 1987). A variety of surgical options exist to treat DORV. Surgeons can use a patch to form a tunnel through the VSD that connects the aorta to the right ventricle, or perform an arterial switch by detaching the aorta from the right ventricle and attaching it to the left ventricle (Walters III et al., 2000).

### Left heart hypoplasia (LHH)

Left heart hypoplasia (LHH), also known as hypoplastic left heart syndrome (HLHS), is a rare manifestation of congenital heart disease (3.8% of cases). However, it causes 25% of all congenital heart disease mortalities (Bradely, 1999), and is one of the most difficult cardiac anomalies to treat. The LHH left ventricle is underdeveloped and unable to support systemic circulation, and LHH is also often associated with mitral and aortic valve anomalies. Infants with LHH may initially be asymptomatic, but become very ill when the ductus arteriosus closes (Tworetzky et al., 2001). Surgery involves bypassing the weak left side of the heart and using the right ventricle for all the pumping by attaching a new aorta to the right ventricle (although oxygen-rich and poor blood still mix in the heart), or creating a bi-directional shunt that allows returning oxygen-poor blood to go directly to the lungs so that the blood does not mix in the heart (Norwood et al., 1980, 1983; Prsa et al., 2010).

## Surgical interventions to alter hemodynamic conditions in chick embryos and consequences

In this section we present the most common surgical interventions performed in chick embryos to alter blood flow conditions. Further, we summarize the changes in hemodynamic conditions associated with the different surgical interventions, changes in cardiovascular tissue properties observed after intervention, and the cardiac defects induced by the intervention.

### Vitelline vein ligation/clipping

Vitelline vein ligation/clipping is a procedure in which one of the vitelline veins that returns blood to the embryonic heart is ligated or clipped, obstructing blood flow (Broekhuizen et al., 1999; Ursem et al., 2004; Stekelenburg-De Vos et al., 2005, 2007). Ligation can be performed using a surgical suture, while clipping uses a surgical clip to obstruct flow. The ligation or clipping can be performed on either the right or left vitelline veins. For this review, we will refer to both VVL and clipping as VVL as they both produce the same hemodynamic alterations. The VVL procedure has been usually performed at HH13–18, during the looping cardiac stages and before cardiac septation begins, when heart formation is sensitive to blood flow conditions.

#### VVL acute hemodynamics

VVL causes blood flow to reroute into the opposite lateral vitelline vein, temporarily reducing hemodynamic load on the developing heart. This manipulation alters the left/right balance of venous inflow to the heart, without chronically changing circulating blood volume. The major hemodynamic changes are mainly acute (lasting only hours after manipulation), but have been suggested to start a cascade of altered growth and remodeling events that lead to defects in the fully formed heart.

**Figure 3:**
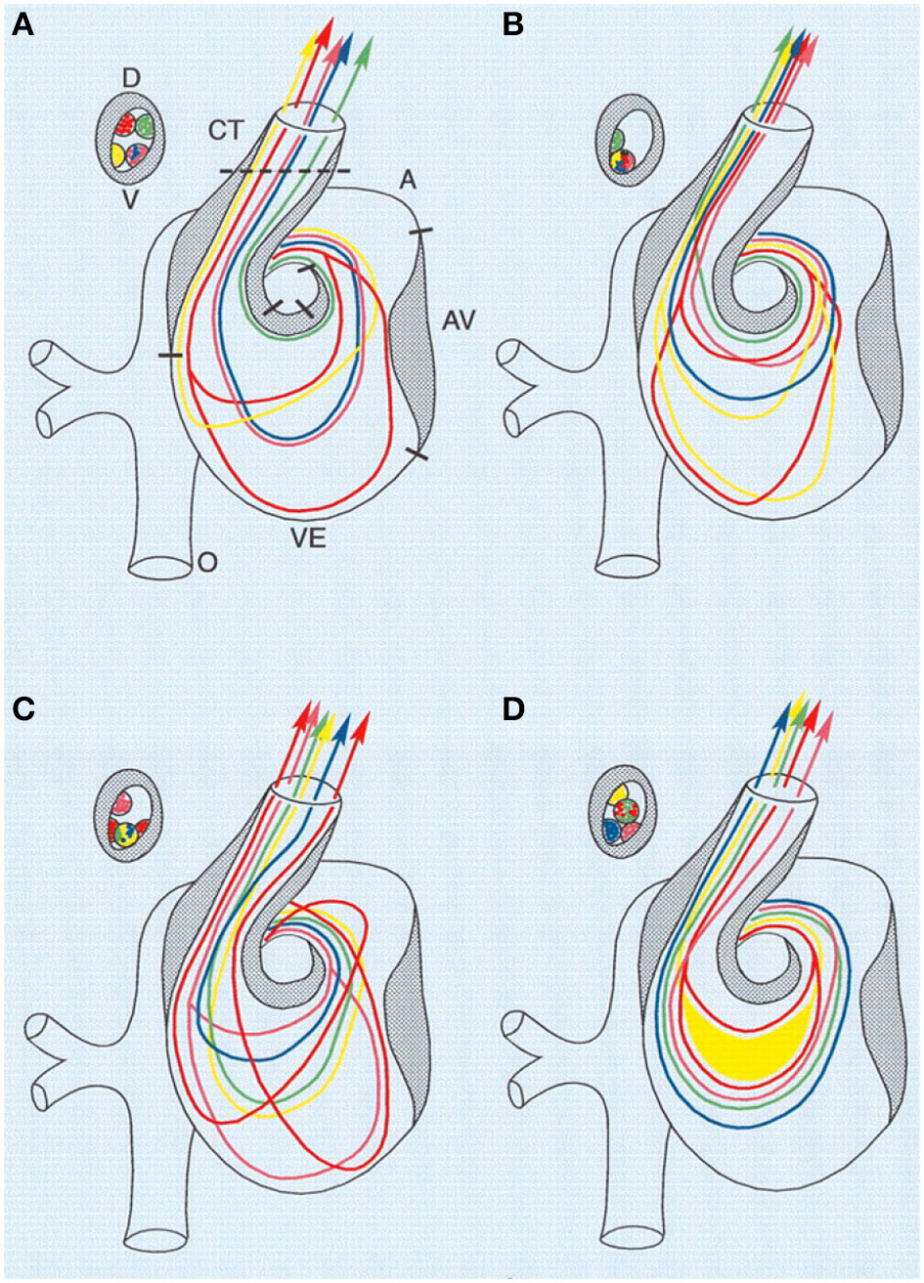
**Combined schematic representation of intracardiac flow patterns after ligation of the different vitelline veins**. Each colored line represents the average flow pattern of one particular yolk sac region. **(A)** Normal flow pattern. **(B)** Flow pattern observed after ligation of the right VVL. There is a reduction of streamlines passing through the left half of the AV canal (red, yellow), and in the OFT all streamlines were located along the lateral-most half and also more ventrally. **(C)** Flow pattern observed after ligation of the left VVL. By crossing from the inner curvature to the outer curvature, the pink streamline is added to the left half of the AV canal. Both red streamlines are sharply edged in the left atrium, ventricular inflow and outflow. **(D)** Flow pattern observed after posterior VVL. Most streamlines were running centrally through the AV canal, with hardly any flow along the outer curvature of the ventricle and the inner curvature of the OFT. Green, anterior yolk sac region; pink, right lateral yolk sac; blue, left lateral yolk sac; yellow, posterior yolk sac, single streamline; red, posterior yolk sac, double streamline. A, atrium; AV, atrioventricular canal; CT, conotruncus, or heart outflow tract; D, dorsal; V, ventral; VE, ventricle. Image reproduced with permission from Hogers et al. (1999).

The altered venous return following VVL has been shown to acutely reduce stroke volume as well as dorsal aortic and outflow tract blood flow velocities (Stekelenburg-De Vos et al., 2003; Rugonyi et al., 2008). After 5 h of right VVL performed at HH17, dorsal aortic peak systolic blood flow velocity was significantly 36% below the peak velocity of the control group, while stroke volume decreased 14% in manipulated embryos compared to controls (Stekelenburg-De Vos et al., 2003). In addition to decreased flow velocities, VVL has also been shown to alter blood flow patterns through the heart. Right VVL caused a small change of the blood flow pattern in the AV canal where the blood flowed more centrally and with shorter streamlines through the left half of the AV canal (as observed in India ink experiments). Left VVL forced blood from the posterior and left lateral yolk sac regions to detour, causing alterations in both the AV canal flow and the inflow portion of the ventricle. Separate from left and right VVL, ligation of the posterior vitelline vein, caused flow changes within the AV canal and ventricle with more central streamlines running through the AV canal (Hogers et al., 1999). See Figure 3. Since endothelial and endocardial cells have responsive elements that are activated by shear stress, cardiac blood flow patterns may be important in regulating normal cardiovascular development (Fisher et al., 2001; Hove et al., 2003; Van Der Heiden et al., 2006). In fact it has been shown that VVL alters gene expression patterns of shear sensitive genes (ET-1, KLF2, eNOS) in the heart endocardium (Groenendijk et al., 2007).

Studies have shown that the acute changes in stroke volume and blood flow velocities subsequently normalize and become comparable to control levels (Broekhuizen et al., 1999; Ursem et al., 2004; Stekelenburg-De Vos et al., 2005, 2007). After at least 24 h of altered flow initiated at HH17, intraventricular end-systolic pressure and end-diastolic pressure are comparable to controls (Stekelenburg-De Vos et al., 2005), and are increased by HH24 (Stekelenburg-De Vos et al., 2007). Pressure-volume loop analysis shows that the increase in pressure at HH24 is accompanied by decreased end-systolic elastance and increased end-diastolic stiffness (Stekelenburg-De Vos et al., 2007). The initial increase in pressure after the acute hemodynamic changes followed by a return to normal levels after 24 h may also impact the final cardiac defects formed.

Little has been reported in the literature about the hemodynamics through the heart following VVL after septation, valve formation and permanent cardiac defects emerge. However, Broekhuizen and colleagues (Broekhuizen et al., 1999) showed that after right VVL at HH17 hemodynamics returned to normal levels by HH24, but then peak systolic blood flow velocity through the dorsal aorta increased by 26% and stroke volume increased by 33% compared to control levels at HH34 (Broekhuizen et al., 1999). Investigators have proposed that an overcompensating Frank-Starling mechanism develops and leads to a more contractile ventricular myocardium. Nevertheless, the observed increase of end-diastolic stiffness at HH24 following VVL manipulation at HH17 (Stekelenburg-De Vos et al., 2007), suggests there may be additional regulatory mechanisms at play.

#### Altered tissue properties following VVL

The acute reduced hemodynamic load induced by VVL has been shown to modify the cardiac tissue architecture in terms of endocardial cell arrangement, ventricular compact layer thickness, and ventricular wall stiffness. These anomalies in the heart architecture become evident after acutely altered hemodynamics have returned to normal levels, showing that the effects of VVL continue beyond the initial few hours.

Endocardial cells align in the direction of flow when wall shear stresses reach a critical level and/or when shear stresses are evenly and constantly distributed along the heart walls in the mature and embryonic cardiovascular systems (Nerem et al., 1981; Icardo, 1989; Davies and Tripathi, 1993; Malek and Izumo, 1996). Following the change in hemodynamic forces induced by VVL, endocardial cell arrangement is altered. Normal endocardial cells in the section of the atrial floor that funnels to the AV canal and dorsal wall of the primitive ventricle align with blood flow by HH27 and appear elongated with smooth surfaces and bulging nuclei. Endocardial cells in the dorsal wall of the right atrium, however, remain unaligned by HH27. After left VVL performed at HH13–14, however, endocardial cell orientation emerges in the dorsal wall of the right atrium by HH27: cells appear elongated and in the orientation of the sinus venosus (Icardo, 1989). These results suggest that the altered blood flow patterns after VVL either increase shear stresses on the dorsal wall of the right atrium or create a more constant shear flow than under normal hemodynamic conditions, which align the endocardial cells.

Since cardiac growth is regulated by hemodynamic load (Clark et al., 1989; Schroder et al., 2002), the acute reduction of load after VVL is thought to delay the normal ventricular growth progression. Histological sections revealed that the ventricles of VVL embryos were less developed than those of controls: the compact layer of the ventricular myocardium was thinner and ventricular trabeculation was reduced in VVL embryos (Hogers et al., 1998). Additionally, Steklenburg-de Vos and colleagues found that end-systolic ventricular elastance was significantly lower in VVL embryos (intervention at HH17) with a 52% decrease by HH21 and 61% decrease by HH24 with respect to controls. This change was accompanied by increasingly higher end-diastolic stiffness at HH21 and HH24 (Stekelenburg-De Vos et al., 2005, 2007). These altered cardiac tissue properties create a less compliant ventricle, which is consistent with normal ventricular tissue at younger developmental stages and suggests that the normal developmental increase in myocardial compliance is also delayed after VVL (Friedman, 1972; Keller et al., 1994; Stekelenburg-De Vos et al., 2005).

#### VVL induced defects

The spectrum of cardiac malformations observed after VVL are hypothesized to stem from impaired heart looping in early developmental stages, and are characterized by increased distance between the inflow and outflow tracts, dextroposed position of the arterial trunk, and disturbed cushion formation (Hogers et al., 1997). These developmental anomalies cause incomplete fusion of septal components leading to VSD. Most cardiac malformations observed with VVL seem to present a VSD and depend on the location and size of the VSD. Frequently a dextroposed or overriding aorta is found, in which the aorta is positioned directly over a VSD rather than over the left ventricle. Even though the VVL manipulation is fairly reproducible, the magnitude of the flow change could still vary from embryo to embryo, and together with biological variations explain why only varying portions of manipulated embryos develop malformations. See **Table 3** for cardiac defect incidence in individual studies.

***Ventricular septal defect.*** VSD is a common defect that develops following VVL, with occurrence varying between reported studies. After right VVL at HH17–18, 10–72% of VVL embryos developed mild to severe VSDs (Oscar, 1965; Hogers et al., 1997, 1999; Broekhuizen et al., 1999). Left VVL also showed VSD formation in 18% of the manipulated embryos (Hogers et al., 1999). VSD is thought to result after VVL from altered hemodynamics and flow patterns that cause the misalignment of septal components (Hogers et al., 1997). In many cases, VSD accompanies other cardiac malformations including aortic arch defects, valve anomalies, and the double outlet right ventricle defect, all of which are discussed in the following sections. Some reports of VSD in early post-septation stages may provide an over estimation of malformations, since a decrease in VSD malformations was shown between stages HH27–35 and HH36–45, when smaller VSDs spontaneously close on their own during development (Hogers et al., 1999).

***Pharyngeal arch artery malformation.*** PAA malformations consistently developed after right VLL at HH17–18 in about a third of the embryos across reported experiments and in 9% of the embryos after left VVL (Hogers et al., 1999). A wide range of anomalies were reported, including arch obliteration, arch interruption, arch persistence, double aortic arch, diameter decrease in the third and sixth arches leading to hypoplastic right brachiocephalic artery, persistent ductus caroticus, and hypoplastic pulmonary artery (Hogers et al., 1997, 1999). Early post-septation stages had more arch malformations combined with VSD, while there were more solitary arch malformations in late post-septation stages (Hogers et al., 1999). One possible contributor to the spectrum of PAA malformations may be due to the fact that VVL surgical manipulation delays and misdirects the neural crest cell migration from the pharyngeal arch region, evidenced by abnormal condensed positioning of the aorticopulmonary septum mesenchyme (Hogers et al., 1997).

***AV valve anomalies.*** Minor AV valve anomalies have been reported following VVL intervention, always accompanied by VSD (Hogers et al., 1997, 1999). About 8% of manipulated embryos showed AV valve malformations after right VVL, in which some valves had the tricuspid opening dorsal to the aorta instead of to the right and some had developmentally immature AV valves (Hogers et al., 1997).

***Semilunar valve anomalies.*** Semilunar valve anomalies are a common defect that develops following VVL. Approximately a third of manipulated embryos develop a semilunar malformed valve accompanied by VSD (Hogers et al., 1997, 1999; Broekhuizen et al., 1999). Following right VVL at HH17, anomalies included an additional valve leaflet and fused commissures on both bicuspid aortic valve leaflets and quadricuspid pulmonary valve leaflets (Hogers et al., 1997, 1999). Left VVL specifically affected the pulmonary valve leaflets (Hogers et al., 1999).

***Double outlet right ventricle.*** DORV defects were observed after both right (aproximately 1/3 of manipulated embryos) and left VVL (Icardo, 1996; Hogers et al., 1997; Broekhuizen et al., 1999). DORV defects were characterized by a dextroposed aorta with a long connection between the base of the aorta and the left ventricle, accompanied by VSD.

### Left atrial ligation

In LAL, a suture is placed around the left atrium and tied in a knot to constrict the left atrioventricular orifice and decrease the effective volume of the left atrium. LAL has been performed at HH21, during the looping stages and before septation commences (Tobita and Keller, 2000; Tobita et al., 2002; Lucitti et al., 2005; Hu et al., 2009).

#### LAL acute hemodynamics

LAL causes reduced cardiac preload and blood volume in the ventricle. As a result of LAL there is less blood to flow through the heart outflow tract and the rest of the circulatory system. The partial ligation of the left atrium reduces its size, narrows the inflow area of the left ventricle, and redirects blood flow from the left to the right side of the heart. The redistributed hemodynamic load results in the underdevelopment of the left side of the heart and compensatory overdevelopment of the right side cardiac structures (Sedmera et al., 1999; Tobita and Keller, 2000).

Preload reduction following LAL has been shown to decrease intraventricular pressure, stroke volume and AV inflow velocities. After LAL at HH21, Tobita and colleagues showed that acute intraventricular peak and end-diastolic pressure decreases in the LAL embryos compared to controls by 11 and 36%, respectively. Pressure remained decreased up to stage HH27, where peak and end-diastolic pressure were each decreased by 18% in manipulated embryos compared to controls (Tobita et al., 2002). Further, maximum AV inflow velocities immediately decreased after LAL at HH21 by 37% and remained decreased at HH25 by 25% (Tobita and Keller, 2000). After LAL at HH21, dorsal aortic stroke volumes also decreased in LAL embryos compared to controls at HH24 (Hu et al., 2009). In contrast, Lucitti and colleagues showed that acute end diastolic, systolic, mean, and pulse pressures measured in the dorsal aorta 1 h after LAL at HH21 were restored and comparable to controls, while stroke volume remained reduced by 55% (Lucitti et al., 2005). By HH24, all dorsal aortic pressure parameters remained similar to controls, while stroke volume was reduced by 33% in LAL embryos compared to controls. Stroke volume in LAL embryos returned to normal levels at HH27 (Lucitti et al., 2005). While it has been shown that arterial pressure increases/decreases with infusion/withdrawal of circulating blood volume (Yoshigi et al., 1996), the relationship following LAL suggests that arterial pressure maintenance (instead of arterial flow/resistance) may be essential for embryo survival and ensured when balancing circulation deficiencies. This highlights the preservative restoration of the embryonic hemodynamic response, and further suggests that differences in acute wall shear stress may be a major factor leading to cardiac defects after LAL.

Little has been reported in the literature about the hemodynamics of blood flow through the heart following LAL after septation, and after permanent cardiac defects occur. However, Tobita and Keller reported blood flow velocities with right and left ventricular wall deformation patterns after inducing LHH with LAL up until HH31. Maximum right ventricular inflow velocities were similar to controls, while average inflow velocities were 14% higher after LAL consistent with increased right ventricular filling volumes. Maximum left ventricle inflow velocity was reduced by 19% in LAL embryos, while average left ventricular inflow velocity was comparable to controls. This demonstrates that hemodynamics remained altered after the major malformations developed, which could induce additional secondary malformations and/or detrimental remodeling.

#### Altered tissue properties following LAL

Chronic reduced preload and ventricular blood volume following LAL have been shown to modify the myocardial architecture prior to the development of cardiac defects. In particular, changes in the myofiber angle distribution, ventricular wall stiffness, compact myocardium thickness, and ventricular dimensions have been documented (Tobita et al., 2002, 2005; Dealmeida et al., 2007; Hu et al., 2009). These alterations are consistent with delayed developmental growth induced by reduced ventricular hemodynamic load.

Decreased left ventricular dimensions prior to full LHH have been observed as early as 1 h after LAL (Dealmeida et al., 2007). While the immediate (within 1 h) change in ventricular dimensions is a mechanical consequence of the decreased blood volume filling the heart, ventricular dimensions of LAL embryos remained smaller compared to controls. After LAL at HH21, HH24 ventricular end-systolic volume decreased by 46% and end-diastolic volume decreased by 45% compared to controls (Hu et al., 2009). Tobita and colleagues reported that left ventricular major axis lengths were also decreased by 6% compared to controls at HH27, while right ventricular major and minor axis lengths remained similar to controls. These ventricular dimensional changes at HH27 were also associated with a 26% decrease in right ventricular compact myocardium thickness, while left ventricular compact myocardium thickness remained similar to controls. Delayed development in the left side of the heart caused by LAL is further supported by increased myocardial wall stiffness, where left ventricular myocardial stiffness at HH27 was 52% larger in experimental embryos (Tobita et al., 2002).

**Figure 4:**
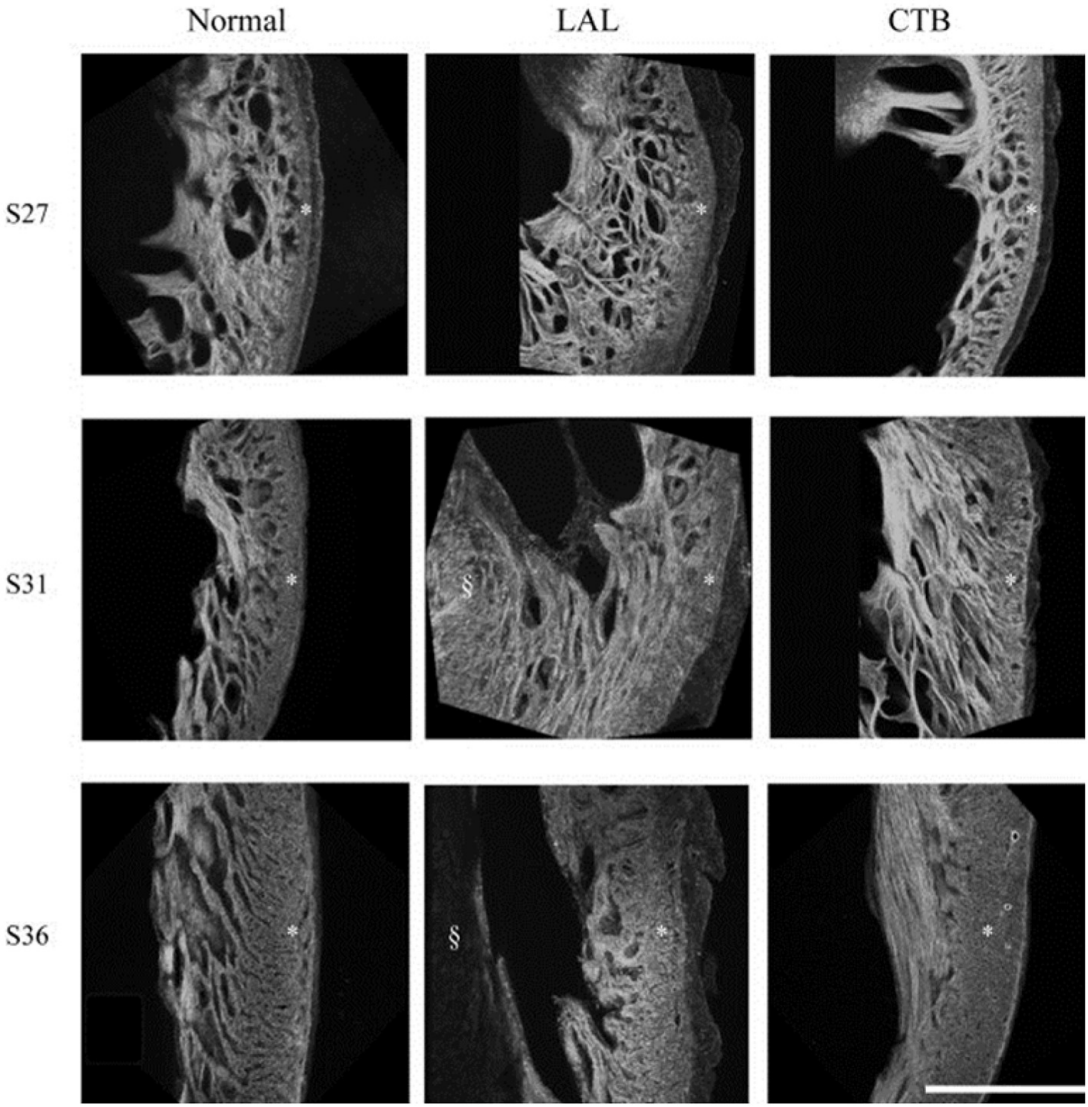
**Developmental change of the LV myocardium architecture in normal, LAL, and OTB embryos shown by 3D reconstructions of f-actin labeled myocardium**. Asterisk, compact myocardium; section symbol, interventricular septum. S27, stage 27 or HH27; S31, stage 31 or HH31; S36, stage 36 or HH36. Scale bar = 500 μm. Image reproduced with permission from Tobita et al. (2005).

Myofiber alignment in the embryonic cardiac wall is an important component in the developmental relationship between cardiac architecture and cardiac function. Myofiber alignment has been shown to change in areas of rapid morphogenesis and proliferation (Itasaki et al., 1989; Shiraishi et al., 1992). During normal development, the myofiber distribution in the left ventricular compact myocardium changes from a uniform circumferential orientation at HH27 to a longitudinal orientation at HH36. After LAL at HH21, myocardial distribution patterns in the left ventricle remained similar to controls at HH27, but were then developmentally delayed and remained circumferentially oriented at HH31 through HH36 (Tobita et al., 2005) (shown in Figure 4). This lack of proper myocardial orientation is associated with altered cardiac function and performance with the underdevelopment of the left side of the heart.

#### LAL induced defects

**Figure 5:**
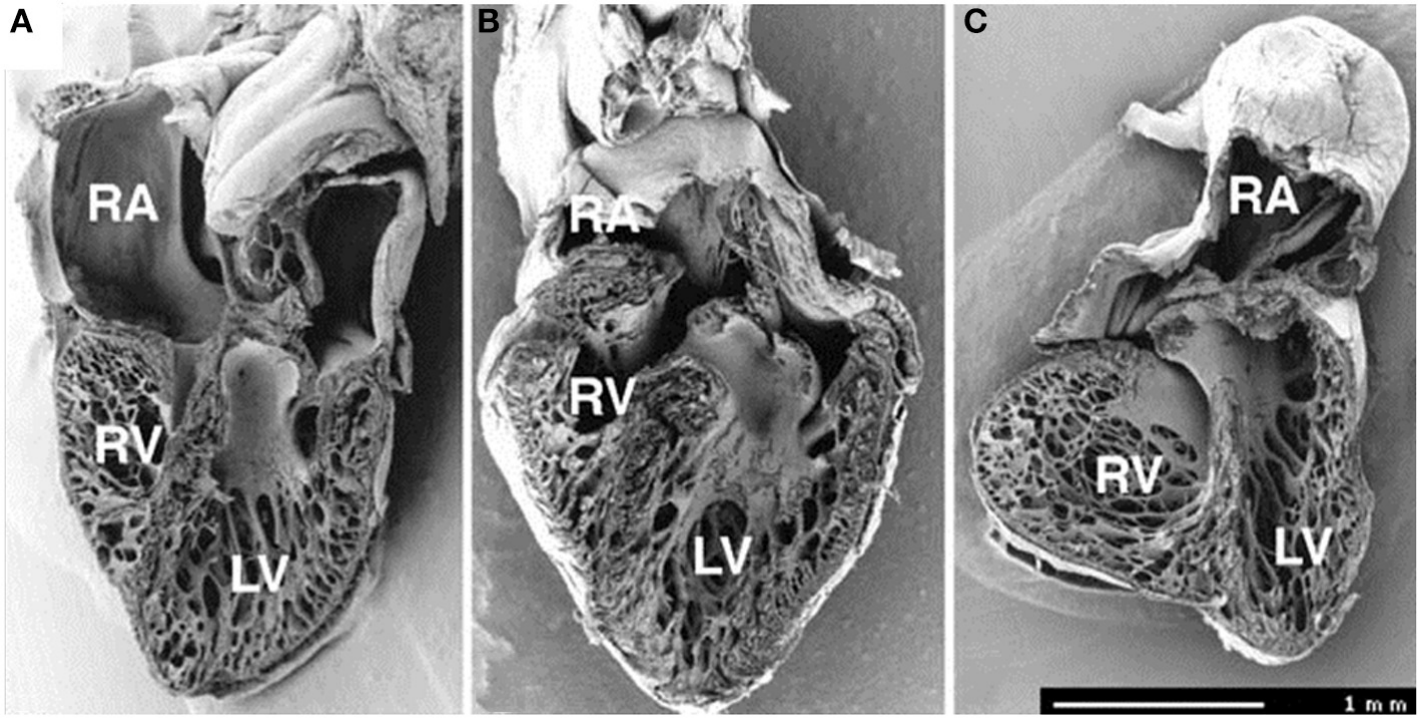
**Frontal dissection of HH34 sham (A), OTB (B), and LAL (C) hearts**. Note distorted atria, changed proportions of the ventricles and defect of interventricular septum in the LAL embryo. The trabeculae in the LV show precocious adherence to the lateral wall and changed orientation with circumferential alignment in the right. The muscular flap-like morphology of the right atrioventricular valve is changed to a bicuspid, mitral-like structure. Ventricular septum defect is seen also in banded heart, where the trabecular compaction is distinctly advanced in both ventricles resulting in thickening of the compact myocardium and interventricular septum and similar modification of right AV valve morphology. Dorsal halves, scale bar = 1 mm. Image reproduced with permission from Sedmera et al. (1999).

Reduced ventricular preload and the associated altered flow patterns induced by LAL interfere with normal looping, septation, and valvular formation to produce cardiac malformations. LAL very reproducibly leads to LHH, which is also associated with varying incidence of VSD, PAA malformation, and abnormal valve morphology. See **Table 3** for defect incidence in individual studies, and Figure 5.

***Left heart hypoplasia.*** LHH is the dominant defect produced by LAL, as redistributed blood flow from the left to the right side of the heart leads to underdevelopment of the left cardiac structures. The LAL model is able to induce LHH very reproducibly, with 100% of manipulated embryos that survive to HH34 displaying a form of the defect (Hu et al., 2009), with the phenotype becoming more pronounced with stage development (Sedmera et al., 1999). After LAL at HH21, the left ventricular area diminished in size by 20% while the right ventricular area enlarged by 40% when compared to controls at HH31 (Tobita and Keller, 2000). Right ventricular transverse diameter has been shown to increase as much as 62% (Harh et al., 1973). Cardiac shape changes following LAL, which highlight LHH, include a rounded apex of the heart that is mostly fully formed by the right ventricle (Harh et al., 1973), decreased left ventricular myocardial volume and accelerated trabecular compaction, while the right side compensation has shown chamber dilatation, and thickening of compact myocardium (Sedmera et al., 1999).

Further analysis of LHH cases generated by LAL has shown modified functional ventricular composition. Vessels in the hypoplastic left side of the heart are developmentally delayed, without lymphatics in the left ventricle (Rychter, 1981). Cell proliferation also decreased in the hypoplastic left ventricle, while the right ventricular trabeculae showed both advanced proliferation and contractile phenotype differentiation compared to the left ventricle (Sedmera et al., 2002).

***Ventricular septal defect.*** VSD was present in about 25% of all LAL hearts (Sedmera et al., 1999). The incidence of VSD following LAL is similar to that of VVL, suggesting that similar hemodynamic factors are at play. Relatively small architectural differences after LAL could be attributed to observed mild VSDs that allow continual intraventricular communication and allow blood to pass to the left ventricle.

***Pharyngeal arch artery malformation.*** About 2/3 of LAL manipulated embryos in reported studies displayed some kind of PAA abnormality. Malformations ranged from abnormal involution patterns, incomplete/interrupted aortic arch formation, asymmetric left side arrangement, smaller left side arterial diameters with twisted appearance, absence of separation between arches (4th and 6th) leading to persistent truncus arteriosus, hypoplastic aortic arch, hypoplastic aortic isthmus, hypoplastic ascending aorta, and patent ductus arteriosus (Harh et al., 1973; Hu et al., 2009). The most common aortic malformation reported was patent ductus arteriosus (Harh et al., 1973).

***AV valve morphology.*** Abnormal AV valve morphology commonly accompanied VSD following LAL manipulations. Irregular remodeling of the right atrioventricular valve produced fibrous bicuspid structures resembling a bicuspid valve more than the normal muscular flap-like morphology of the AV valve, the extent of which was related to the degree of left ventricular hypoplasia. Extreme cases also showed dysplastic (thickened) or atretic (completely closed) left atrioventricular valves (Sedmera et al., 1999). Tobita and Keller also noted that after LAL at HH21, the right AV valve appeared larger than the left AV valve at HH31 (Tobita and Keller, 2000). These observations indicate that hemodynamic loading is an important determinant in the development of valvular structures.

***Semilunar valve morphology.*** Harh and colleagues reported that stenotic (thickened) aortic valves combined with hypoplastic ascending aorta was evident in 15% of LAL manipulated embryos. Normal valve formation results when endocardial cushion swelling forms a dome-shaped ridge at the primitive site of the valve, which later differentiates into the thin, mature valvular layer. Hypoplastic stenotic aortic valves are thought to result when the early differentiation fails and the valve tissue does not thin (Harh et al., 1973). These anomalies and delayed development are attributed to the altered blood flow conditions introduced by LAL.

### Outflow tract banding

In OTB, a suture is placed around the heart outflow tract and tied to constrict the lumen diameter. OTB has been performed at HH18-HH21, during the looping cardiac stages, by several groups (Clark and Rosenquist, 1978; Clark et al., 1989; Sedmera et al., 1999; Tobita et al., 2002, 2005; Miller et al., 2003; McQuinn et al., 2007; Rugonyi et al., 2008; Shi et al., 2013).

#### OTB acute hemodynamics

OTB constricts/decreases the lumen diameter of the outflow tract so that hemodynamic load is increased in the heart ventricle. Unlike LAL, which decreases cardiac workload on the early embryonic heart, OTB increases cardiac workload on the early heart tube. Banding generates increased blood pressures throughout the embryonic cardiovascular system, and increased blood flow velocity in the region of the band. After OTB, hemodynamics are quickly altered and remain changed throughout development. Peak and end-diastolic intraventricular pressures immediately increase following OTB (Tobita et al., 2002; Shi et al., 2013). After OTB at HH21, peak ventricular pressure increased 36%, and end-diastolic pressure increased 11% compared to controls (Tobita et al., 2002). Not only does blood pressure increase after banding, but the extent of the increase also depends on band tightness. Shi and colleagues found that both diastolic blood pressure (minimum pressure) and pulse pressure amplitude (and therefore systolic pressure) in the ventricle, aortic sac, and dorsal aorta increased somewhat linearly with band tightness until near 40% band constriction, at which point pressure measures started to increase faster than linearly with band tightness (Shi et al., 2013). Differences in hemodynamic response caused by band tightness may be partly responsible for the spectrum of cardiac defects seen after cardiac septation is complete.

Intraventricular pressures remained higher in OTB embryos days after manipulation. After banding at HH21, blood pressure was reported through stage HH27 (Clark et al., 1989; Tobita et al., 2002) when peak and end-diastolic intraventricular pressures were 21 and 27% higher than controls, respectively (Tobita et al., 2002). Increased pressures were accompanied by increased peak blood flow velocities near the band site (and presumably increased wall shear stresses on the lumen walls) (McQuinn et al., 2007; Rugonyi et al., 2008). Flow velocities close to the band increased as much as 120% through the OFT (McQuinn et al., 2007).

#### Altered tissue properties following OTB

Overload induced by OTB has been shown to modify the myocardial architecture prior to the development of cardiac defects. OTB alters cardiac ventricular dimensions, ventricular compact layer thickness, ventricular mass, ventricular stiffness, and myofiber angle distribution. Ventricle dilation occurs immediately after constricting the OFT lumen walls, first as a passive response to increased blood pressure but then through growth and remodeling, as the increased dimensions are sustained until septation (Sedmera et al., 1999; Tobita et al., 2002). Since banding causes increased resistance to flow at the site where the band is placed, the heart approaches a more “ideal” spherical shape for efficient pumping with changes in both the right and left ventricles (Hutchins et al., 1978). Tobita et al. showed that after OTB at HH21, right ventricle major and minor axis lengths increased by 14 and 17% compared to controls at HH27, respectively, while left ventricle minor axis lengths increased by 10% compared to controls at HH27 and major axis length remained comparable to controls (Tobita et al., 2002). The increased hemodynamic load imposed on the heart by OTB increased the rate of ventricular growth without changing the overall rate of embryo growth or morphogenesis of the ventricle. Clark et al. showed that the increased ventricular mass is due to myocyte hyperplasia (proliferation), since organelle ratios, DNA-to-total protein ratio, and myocyte areas are constant among experimental and control groups (Clark et al., 1989). Trabecular compaction in OTB embryos was more advanced than in controls, with increased ventricular compact layer thickness as early as 12 h after OTB (Sedmera et al., 1999), and up to HH27(Tobita et al., 2002). Trabeculae also exhibits a spiraled and dense pattern in OTB samples that is not seen in controls, and this spiral pattern is speculated to be part of a mechanism to increase the pumping efficiency of the pressure-overloaded embryonic heart (Sedmera et al., 1999). Miller and colleagues reported that passive left ventricular stiffness almost doubled by HH27 after OTB at HH21, with longer exposure to overload resulting in larger stiffness increases. While ventricular compliance normally increases over development, the increased stiffness after OTB may be a result of tissue remodeling to offset increased wall strain induced by OTB (Miller et al., 2003).

Myofiber alignment is also affected by the banding procedure. OTB produces the opposite myofiber angle distribution response compared to LAL (in which maturation toward normal myocardial distribution patterns is delayed). After OTB at HH21 the myofiber distribution in the compact myocardium remained similar to controls at HH27. Later, myofiber maturation patterns accelerated at stages HH31–36 (Tobita et al., 2005) with myofiber alignment changing rapidly to a longitudinal orientation characteristic of normal HH36 embryos. See Figure 4 to compare interventions. These results suggest that myocardial alignment may be regulated by blood pressure: when blood pressure is low (LAL) alignment is delayed; when blood pressure is high (OTB) alignment is accelerated. During normal development, ventricular blood pressure constantly increases, and this increase is perhaps used in the regulation of myofiber alignment.

#### OTB induced defects

Increased ventricular afterload and the associated flow patterns induced by OTB interfere with normal looping, septation, and valvular formation to produce cardiac malformations. OTB produces a wide spectrum of defects with varying incidence depending on the report. These cardiac malformations include VSD, PAA malformation, abnormal valve morphology, and double outlet right ventricle. See **Table 3** for defect incidence in individual studies, and Figure 5.

***Ventricular septal defect.*** VSD is commonly seen after OTB, with incidence ranging from 76 to 100% in manipulated embryos (Clark and Rosenquist, 1978; Sedmera et al., 1999), compared to only ~25% of all LAL hearts developing VSD (Sedmera et al., 1999). Similar to the other surgical interventions, VSD associated with OTB is often combined with other defects including DORV and PAA defects (Clark et al., 1984; Sedmera et al., 1999). Total incidence of VSD formation is directly related to duration of OTB, and increases with longer band placement. Clark and Rosenquist demonstrated that VSD malformation incidence increased with banding duration where after banding for 2, 6, 24 h, and permanently, incidence was 0, 44, 100, and 76%, respectively (please note, however, that number of embryos in each group varied, in part due to variability in survival rates) (Clark and Rosenquist, 1978). Malformation later in development after only 6 h of OTB, indicates that hemodynamics play an essential role in regulating growth and morphogenesis and that it is unlikely that cardiac defects result solely from the physical placement of the suture.

***Pharyngeal arch artery malformation.*** A wide spectrum of PAA abnormalities has been observed after OTB. Clark and Rosenquist reported that 56–100% of OTB embryos developed aortic arch malformation depending on the duration of outflow tract constriction. Malformations included complete absence, interruption, and tubular hypoplasia, and were always associated with VSD (Clark and Rosenquist, 1978). Persistent truncus arteriosus, one of the most severe PAA defects, has also been reported following OTB but not in VVL or LAL (Sedmera et al., 1999).

***AV valve morphology.*** Abnormal AV valve morphology following OTB shows that loading is an important determinant in valvular development. While incidence was not reported, in some cases, right AV valve morphology changed from normal muscular flap-like to bicuspid valve, and became more similar to the mitral valve (Sedmera et al., 1999).

***Double outlet right ventricle.*** DORV was also observed after OTB, although incidence was not reported. Cardiac defects induced by OTB are usually associated with alignment complications of the outflow tract during the looping stages. Looping problems in early development are thought to result in a modified orientation of the aortic and mitral valves, when the separation between the annuli is increased. Severe separation cases can result in DORV, with both great vessels arising from the right ventricle (Clark et al., 1984; Sedmera et al., 1999).

## Conclusions and future suggestions

The studies reviewed in this article show that surgical interventions that alter hemodynamics in the early chick embryo result in cardiac tissue property changes and produce a wide spectrum of cardiac defects. The main hemodynamic interventions performed in chick embryos are VVL, LAL, and OTB, and they are typically performed during the cardiac looping stages (HH13–21). Further, these interventions are complementary, with VVL and LAL decreasing blood pressure and flow, and OTB increasing blood pressure and flow velocities. Cardiac defect formation is likely very dependent on when the surgical manipulation is performed (stage of development) and what specific developmental processes are interrupted. Tables 1–3 summarize outcomes resulting from various embryo manipulations; please refer to the text for specific details. While the stage at which interventions were performed in individual studies varied, the majority of the reviewed manipulations was performed between HH17 and HH21, during the final normal stages of looping and before initial stages of septation and valve formation, and thus results should be comparable. The three surgical interventions discussed result in many of the same cardiac defects, which indicate that the altered hemodynamics interfere with common looping, septation and valve formation processes that occur after intervention and that shape the four-chambered heart. While many similar defects develop after the interventions, the varying degrees of hemodynamic load alteration among the three interventions also result in varying incidence and severity of cardiac defects. Investigations of the effects VVL, LAL, and OTB have all reported VSD, PAA malformations, and valve anomalies. LHH was only reported in LAL, and DORV was only reported in VVL and OTB (see also Table 3). These results seem to indicate that the hemodynamic modulation of cardiac developmental processes is strongly dependent on hemodynamic load.

**Table 1:** **Summary of reported hemodynamics after surgical intervention**.

**Surgical intervention**	**DA End-systolic pressure**	**DA End-diastolic pressure**	**Ventricular end-systolic pressure**	**Ventricular end-diastolic pressure**	**Stroke volume**	**DA blood velocity**	**AV canal blood velocity**	**OFT blood velocity**
VVL	–	–	–	–	↓(acute)	↓(acute)	–	↓(acute)
	Broekhuizen et al., 1999	Broekhuizen et al., 1999	Stekelenburg-De Vos et al., 2005	Stekelenburg-De Vos et al., 2005	Stekelenburg-De Vos et al., 2003	Stekelenburg-De Vos et al., 2003	Ursem et al., 2004	Rugonyi et al., 2008
			↑	↑	–	–		
			Stekelenburg-De Vos et al., 2007	Stekelenburg-De Vos et al., 2007	Broekhuizen et al., 1999; Ursem et al., 2004; Stekelenburg-De Vos et al., 2005, 2007	Broekhuizen et al., 1999		
LAL	–	–	↓	↓	↓		↓	
	Lucitti et al., 2005	Lucitti et al., 2005	Tobita et al., 2002	Tobita et al., 2002	Hu et al., 2009; Lucitti et al., 2005		Tobita and Keller, 2000	
OTB	↑	↑	↑	↑	–			↑
	Shi et al., 2013	Shi et al., 2013	Clark et al., 1989; Tobita et al., 2002; Shi et al., 2013	Clark et al., 1989; Tobita et al., 2002; Shi et al., 2013	McQuinn et al., 2007			McQuinn et al., 2007; Rugonyi et al., 2008

Interventions were performed between HH17-HH21, and reported hemodynamic conditions were measured between HH21 and HH27 (except for noted acute measurements). References correspond to reported change.

↓ Decrease in hemodynamic measure in experimental group compared to controls.

– No significant change in hemodynamic measure in experimental group compared to controls; ↑ Increase in hemodynamic measure in experimental group compared to controls.

**Table 2:** **Summary of reported altered material properties after surgical interventions**.

**Surgical intervention**	**Increased ventricular dimensions**	**Decreased ventricular dimensions**	**Decreased ventricular compact layer thickness**	**Increased ventricular compact layer thickness**	**Increased ventricular wall stiffness**	**Increased ventricular mass**	**Accelerated myofiber angle distribution**	**Delayed myofiber angle distribution**	**Abnormal endocardial cell arrangement**
VVL			Hogers et al., 1998		Stekelenburg-De Vos et al., 2005, 2007				Icardo, 1989
LAL		Tobita et al., 2002;Dealmeida et al., 2007; Hu et al., 2009	Tobita et al., 2002		Tobita et al., 2002			Tobita et al., 2005	
OTB	Sedmera et al., 1999; Tobita et al., 2002			Sedmera et al., 1999; Tobita et al., 2002	Miller et al., 2003	Clark et al., 1989	Tobita et al., 2005		

Only includes properties that were observed prior to HH36, before the heart has septated chambers and valves. References correspond to reported change.

**Table 3:** **Summary of reported cardiac defects after surgical intervention**.

**Surgical intervention**	**Ventricular septal defect**	**Pharyngeal arch artery malformation**	**Atrioventricular valve malformation**	**Semilunar valve malformation**	**Double outlet right ventricle**	**Left heart hypoplasia**
VVL	**R:10–72%, L:18%**	**R:~35%, L:9%**	**R:8%**	**R:~25%**	**R:~35%**	
	(Hogers et al., 1997) R 57% (Hogers et al., 1999) R10%, L18% (Broekhuizen et al., 1999) R72% (Oscar, 1965)	(Hogers et al., 1997) R35% (Hogers et al., 1999) R32% L9% (Broekhuizen et al., 1999) R33%	(Hogers et al., 1997) R8% (Hogers et al., 1999)	(Hogers et al., 1997) R21% (Hogers et al., 1999) R34% L36% (Broekhuizen et al., 1999) R39%	(Hogers et al., 1997) R37% (Broekhuizen et al., 1999) R33% (Icardo, 1996)	
LAL	**25%**	**~66%**	**X**	**15%**		**62–100%**
	(Sedmera et al., 1999) 25%	(Hu et al., 2009) 70% (Harh et al., 1973)^*^ 62%	(Tobita and Keller, 2000) (Sedmera et al., 1999)	(Harh et al., 1973)^*^ 15%		(Harh et al., 1973)^*^ 62% (Hu et al., 2009) 100% (Sedmera et al., 1999, 2002; Tobita and Keller, 2000; Tobita et al., 2005; Dealmeida et al., 2007; Rychter, 1962)
OTB	**76–100%**	**56–100%**	**X**		**X**	
	(Sedmera et al., 1999) 100% (Clark and Rosenquist, 1978) 76–100% (Clark et al., 1984)	(Clark and Rosenquist, 1978) 56–100% (Sedmera et al., 1999)	(Sedmera et al., 1999)		(Clark et al., 1984; Sedmera et al., 1999)	

References correspond to reported percentage of embryos with defect.

* Obstruction of the left AV canal using suture material- same effect as LAL; R, right VVL; L, left VVL.

**X**, Incidence was not reported.

Very little has been reported about the hemodynamic conditions of fully developed hearts. Since hemodynamics has been proven fundamental in early development, future investigations should also focus on the effect of sustained hemodynamic alterations in the heart to fully characterize the malformed cardiac deficiencies. In this context, it should also be recognized that even cases in which cardiac malformations originate from disparate factors (e.g., genetic anomalies, teratogen exposure, etc.), the altered blood flow patterns that emerge will concomitantly affect cardiac growth, remodeling and morphogenesis. With the same logic, factors that affect blood flow (e.g., by modifying normal cardiac contractility during development) might result in cardiac malformations that are due to flow conditions. Altered flow, in addition, will lead to secondary malformations in the vasculature and changes in tissue properties throughout the cardiovascular system. It is therefore difficult to separate the effect of hemodynamics from the effects of other factors. All aspects (hemodynamics, genetics, exposure) should be considered when evaluating the causes of congenital heart malformations, and when devising treatment options for infants with congenital heart disease.

Little is known about non-genetic mechanisms of cardiac malformation, and in particular the mechanisms by which blood flow modulates cardiac development. Early stages of cardiac malformation and the consequences of altering blood flow need to be more thoroughly investigated. To this end, research that integrates hemodynamic data with biological change in response to hemodynamics, and the mechanisms behind this response, are essential. Several studies to determine hemodynamics in the developing embryonic heart are have been recently published (McQuinn et al., 2007; Stekelenburg-De Vos et al., 2007; Liu et al., 2012; Kowalski et al., 2013; Shi et al., 2013), as well as studies to look at remodeling and changes in tissue properties shortly after interventions and flow exposure (Groenendijk et al., 2007; Biechler et al., 2010; Yalcin et al., 2011; Buskohl et al., 2012; Tan et al., 2013). As these two aspects of cardiac development are put together, a more complete picture of heart formation is emerging where developmental programming plays a substantial role, which needs to be better characterized and studied. Understanding how hemodynamics determines cardiac fate early on will certainly provide clues to design better treatment options and strategies for babies with cardiac malformations.

### Conflict of interest statement

The authors declare that the research was conducted in the absence of any commercial or financial relationships that could be construed as a potential conflict of interest.

## Abbreviations

AV: atrioventricular
DORV: double outlet right ventricle
LAL: left atrial ligation
OFT: outflow tract
OTB: outflow tract banding
VSD: ventricular septal defect
VVL: vitelline vein ligation
